# Outcomes of Trabeculectomy With and Without Mitomycin C in Pseudoexfoliative Glaucoma Compared With Mitomycin C in Primary Open Angle Glaucoma

**Published:** 2019

**Authors:** Ana Sofia LOPES, Fernando Trancoso VAZ, Susana HENRIQUES, Maria LISBOA, Cristina VENDRELL, Isabel PRIETO

**Affiliations:** 1 Ophthalmology Department, Professor Doutor Fernando Fonseca Hospital (HFF), Lisbon, Portugal

**Keywords:** Pseudoexfoliative Glaucoma, Pseudoexfoliative Syndrome, Risk Factor, Trabeculectomy, Mitomycin C, Primary Open Angle Glaucoma

## Abstract

The aim of this study was to evaluate the outcomes of trabeculectomy with mitomycin C (MMC) in patients with Pseudoexfoliative Glaucoma (PXG) and compare the results with the outcomes of trabeculectomy without MMC in PXG and with MMC in Primary Open Angle Glaucoma (POAG). Ninety eyes (76 patients) submitted to trabeculectomy were included in a one-year retrospective study. Fifty-eight eyes with PXG were divided into group 1 (28 eyes) and group 2 (30 eyes), with and without MMC application respectively. Then, the group 1 results were compared with 32 eyes with POAG that performed trabeculectomy with MMC (group 3). Main outcome measures were intraocular pressure (IOP), number of IOP lowering medications, rate of bleb failure (encapsulation, flattening and/or vascularization) and the number of eyes submitted to surgical procedures after trabeculectomy (needling, 5-fluorouracil (5FU) or 2nd trabeculectomy). Results revealed that compared to trabeculectomy with MMC in POAG and trabeculectomy with MMC in PXG, trabeculectomy without MMC in PXG leads to higher IOP (preoperative mean ± standard deviation [SD] was 28.6 ± 5.4 mmHg in group 1, 32.2 ± 8.2 mmHg in group 2 and 26.1 ± 6.5 mmHg in group 3; and after one year was 13.9 ± 3.9 mmHg in group 1, 16.1 ± 5.9 mmHg in group 2 and 12.5 ± 4.0 mmHg in group 3); higher number of IOP lowering medications (preoperative mean ± SD was 3.1 ± 0.60 in group 1, 2.8 ± 0.81 in group 2 and 3.4 ± 0.76 in group 3; and after one year was 1.1 ± 1.1 in group 1, 1.1 ± 1.0 in group 2 and 0.33 ± 0.89 in group 3); higher prevalence of bleb failure (47% in group 1, 53% in group 2, and 18% in group 3); and increased participation in surgical procedures following trabeculectomy (47% in group 1, 57% in group 2, and 6% in group 3). We concluded that trabeculectomy without MMC in PXG had the worst surgical outcome. Thus, PXG appears to be a potential risk factor for filtration bleb failure. Therefore, it could be considered in surgical protocols of MMC application.

## INTRODUCTION

Pseudoexfoliative glaucoma (PXG) is the most usual form of secondary open-angle glaucoma, which develops as a result of pseudoexfoliative syndrome (PXS), a systemic circumstance characterized by abnormal production and accumulation of extracellular elastin-related microfibrillar materials within the anterior segment of the eye (including the lens capsule, ciliary body, iris, zonules and trabecular meshwork) and other organs such as the heart, lungs and kidneys. In contrast to primary open angle glaucoma (POAG), it has a more aggressive clinical course with higher mean intraocular pressure (IOP), peak IOP, wider IOP fluctuation, as well as severe visual field damage and greater optic disc damage at diagnosis, faster rates of progression with more rapid visual field deterioration, worse response to medical therapy and greater need of surgical intervention [1-6].

Both genetic and environmental factors seem to show a role in PXS pathogenesis, and specifically, it has been associated with polymorphisms in the lysyl oxidase-like protein 1 (LOXL1) gene, an important gene for elastin metabolism. The main mechanisms of glaucoma in PXS are the blockage of the trabecular meshwork by exogenous and endogenous exfoliation materials and trabecular meshwork dysfunction. Moreover, the following factors are well-documented characteristics of PXS eyes and possible triggering factors of the abnormal matrix process in tissue of the anterior segment in PXS: increased oxidative stress conditions, a pronounced anterior chamber hypoxia, elevated homocysteine levels, increased transforming growth factor beta (TGF-β) and endothelin-1 levels in aqueous and a transient upregulation of proinﬂammatory cytokines such as interleukin (IL)-6 and IL-8. PXG has also been reported to be associated with more severe destruction of the blood-aqueous barrier after trabeculectomy than POAG, as well as with iris vasculopathy and systemic vascular diseases. These changes contribute to a greater inflammation profile and probably affect surgical outcomes after trabeculectomy [1-9].

Trabeculectomy has become the gold standard of glaucoma surgery since its presentation in 1967 [10]. Nevertheless, long-term cumulative failure rates of trabeculectomy are associated with progressive fibrosis at the episcleral-conjunctival interface with bleb scarring. Adjunctive antimetabolites, such as 5-fluorouracil (5FU) and mitomycin C (MMC), are commonly used to enhance success after trabeculectomy, and its benefits have been demonstrated in several clinical trials, as they reduce subepithelial fibrosis [10-14]. The MMC is a potent non-specific alkylating agent whose active metabolite would crosslink DNA molecules and thereby inhibit DNA synthesis, acting on fibroblasts and endothelial cells [10-14]. However, there is still no standard protocol regarding the dose and duration of MMC application during trabeculectomy, as well as controversy regarding the delivery vehicle and the size or shape of the sponge [10, 15-17].

The authors decided to evaluate the outcomes of trabeculectomy with MMC in PXG and compare the results with the outcomes of trabeculectomy without MMC in PXG and with MMC in POAG. The aim was to analyze the PXG as a risk factor for trabeculectomy failure. 

## METHODS

The surgical technique applied was the modified trabeculectomy of the Moorfields Safer Surgery System (MSS), developed by Peng Khaw and colleagues in 2005. The choice of MMC was stratified according to risk factors for surgical failure, and to achieve this purpose we applied MMC according to the Moorfields Eye Hospital/Florida University Risk Classification and the European Glaucoma Society (EGS) Guidelines (Tables 1 and 2) [14,18]. Accordingly, and to better compare the groups, all the patients were included in the same group risk (high group risk) submitted to the same dose and duration of MMC, and as such were submitted to the application of MMC at a dose of 0.4 mg/mL for 3 minutes, immediately followed by profuse washing of the contact surface with saline solution.

**Figure 1:**
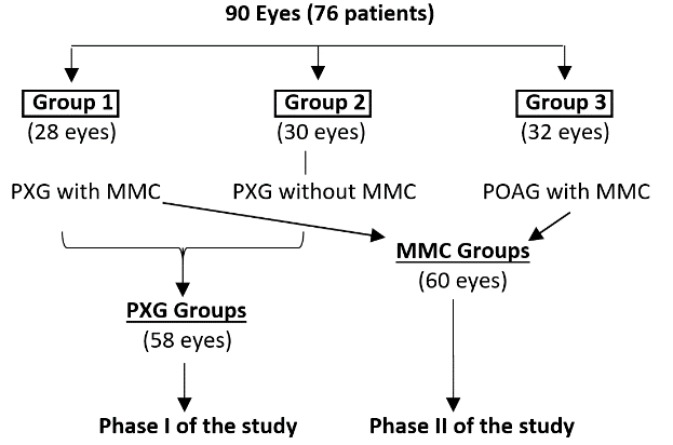
Study Design Diagram

Abbreviations: PXG: Pseudoexfoliative glaucoma; MMC: mitomycin C; POAG: Primary open angle glaucoma.

Ninety eyes of 76 patients undergone modified trabeculectomy (MSS) from January 2014 to March 2018 were included in a retrospective study with a 12-month follow-up, performed at a tertiary single-center (Department of Ophthalmology, Professor Doutor Fernando Fonseca Hospital, Lisbon, Portugal). Exclusion criteria were any type of glaucoma other than POAG and PXG, aphakia, history of corneal or retinal surgery with a conjunctival approach and follow-up episode not totaling 12 months. The eyes were allocated to three groups (Fig 1) – group 1 (28 PXG eyes with MMC application), group 2 (30 PXG eyes without MMC application) and group 3 (32 POAG eyes with MMC application). The same experienced surgeon performed all surgeries. Main outcome measures were IOP, the number of anti-glaucomatous medications (active principles), rate of bleb failure (encapsulation, flattening and/or vascularization) and the number of eyes submitted to surgical procedures after trabeculectomy (needling, 5FU or second trabeculectomy). The patients were observed preoperatively and 1, 3, 6 and 12 months postoperatively.

The study had two phases (Fig 1): in the first one, group 1 was compared with group 2 (PXG groups), and in the second phase group 1 was compared with group 3 (MMC groups). Data was collected from each patient’s medical records. This study followed the tenets of the Declaration of Helsinki. Ethical approval received from a related academic institution and all patients signed a written informed consent after a detailed explanation of the study. Statistical analysis was performed with SPSS 22.0 software (SPSS Inc., Chicago, Illinois, USA). Results were considered statistically significant if the p-value was equal to or less than 0.05. Statistical tests applied were T-Student, Chi-Square, Shapiro-Wilk, Wilcoxon and Mann-Whitney.

**Table 1:** Moorfields Eye Hospital/Florida University Risk Classification. Abbreviations: 5FU: 5-fluorouracil; MMC: Mitomycin C; mg: milligram; mL: milliliter [14,18].

Low risk	Moderate risk	High risk
Nothing or 5FU 50mg/mL in 5 minutes	**5FU 50 mg/mL in 5 minutes or MMC 0.2 mg/mL in 3 minutes**	**MMC 0.4 mg/mL in 3 minutes**
**Without risk factors**	Topic therapy (epinephrine)	Neovascular glaucoma
**Topic therapy (β-antagonists or pilocarpine)**	Previous cataract surgery (without conjunctival incision and capsule rupture)	Chronic persistent uveitis
**Young (< 40 years) without other risk factors**	Several low-risk factors	Previous trabeculectomy failure
**Black elderly**	Phacotrabeculectomy	Chronic conjunctival inflammation
**-**	Previous conjunctival surgery (as in strabismus, retinal detachment, and trabeculectomy)	Multiple risk factors
**-**		Aphakic glaucoma

**Table 2:** European Glaucoma Society (EGS) Guidelines for Risk Factors for Trabeculectomy Failure. Abbreviations: Afro-Caribbean race: African-Caribbean race [14,18].

Neovascular glaucoma
Previous trabeculectomy failure
Former cataract surgery with the conjunctival incision
Aphakia (intracapsular cataract surgery)
Current surgery (< 3months)
Inflammatory ocular disease
Hispanic or Afro-Caribbean race
Young age
Long term use of topical medication

## RESULTS

Ninety eyes of 76 patients with mean ± standard deviation (SD) age of 64.0 ± 11.4 years; 40 females and 36 males included in this study. 

**Figure 2:**
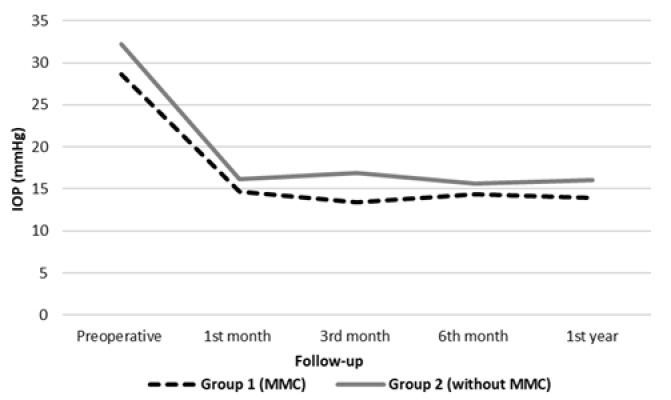
Evolution of Intraocular Pressure (IOP) in PXG Groups (group 1 – PXG with MMC, and group 2 – PXG without MMC). Abbreviations: PXG: Pseudoexfoliative glaucoma; MMC: mitomycin C.

**Figure 3:**
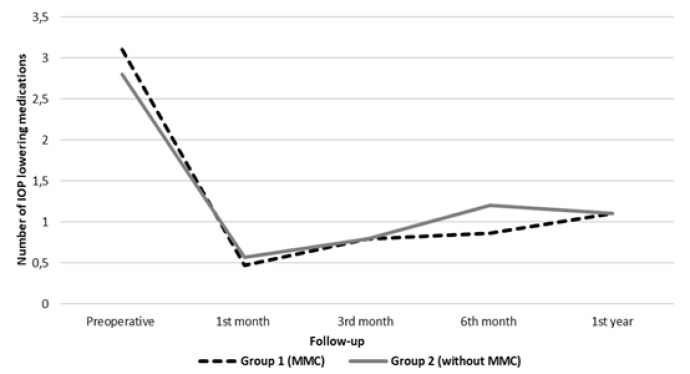
Evolution of the Number of IOP Lowering Medications in PXG Groups (group 1 – PXG with MMC, and group 2 – PXG without MMC). Abbreviations: PXG: Pseudoexfoliative glaucoma; MMC: mitomycin C.

PHASE I – Comparison between PXG groups (group 1 – with MMC, and group 2 – without MMC), Table 3:

1. Evolution of IOP (Fig 2): mean ± SD of preoperative IOP was 28.6 ± 5.4 mmHg in group 1 and 32.2 ± 8.2 mmHg in group 2. The mean ± SD IOP at the 1st month was 14.7 ± 5.8 mmHg (group 1) and 16.2 ± 6.8 mmHg (group 2); at the 3rd month 13.4 ± 4.1 mmHg (group 1) and 16.9 ± 6.4 mmHg (group 2); at the 6th month 14.4 ± 5.3 mmHg (group 1) and 15.6 ± 4.3 mmHg (group 2); and at the 1st year 13.9 ± 3.9 mmHg (group 1) and 16.1 ± 5.9 mmHg (group 2). The group 1 had lower IOP values at all stages of follow-up, but a statistically meaningful difference of IOP reduction noted between the two groups only at the 3rd month after the surgery (P = 0.04). The difference between preoperative IOP and at the 1st year was statistically significant in both groups (P = 0.001 in both).

2. Evolution in number of medications (Fig 3): mean ± SD of preoperative number of medications were 3.1 ± 0.60 (group 1) and 2.8 ± 0.81 (group 2); at the 1st month 0.47 ± 0.64 (group 1) and 0.57 ± 0.85 (group 2); at the 3rd month 0.79 ± 1.0 (group 1) and 0.79 ± 0.89 (group 2); at the 6th month 0.86 ± 1.1 (group 1) and 1.2 ± 1.1 (group 2); and at the 1st year 1.1 ± 1.1 (group 1) and 1.1 ± 1.0 (group 2). Group 1 had less medication through the follow-up and no statistically significant difference in medications number was found between the two groups. The difference between preoperative IOP and at the 1st year was statistically significant in both groups (p = 0.001 in group 1 and P = 0.0017 in group 2).

3. Bleb failure: group 1 had a lower prevalence of bleb failure than group 2 (47% versus 53%, p = 0.045).

4. Additional surgical procedures performed after trabeculectomy: additional surgical procedures were performed more often in group 2 (57% versus 47%, P = 0.04).

**Table 3:** Comparison Between Groups 1 and 2 (group 1 – PXG with MMC, and group 2 – PXG without MMC).

	Group 1 (PXG with MMC)	Group 2 (PXG without MMC)	Significance of the difference
IOP	Mean± SD	Mean± SD	P value
** Preoperative **	28.6 ± 5.4	32.2 ± 8.2	0.102
** 1** ^st^ ** month **	14.7 ± 5.8	16.2 ± 6.8	0.186
** 3** ^rd^ ** month **	13.4 ± 4.1	16.9 ± 6.4	**0.04**
** 6** ^th^ ** month **	14.4 ± 5.3	15.6 ± 4.3	0.887
** 1** ^st^ ** year**	13.9 ± 3.9	16.1 ± 5.9	0.107
** Difference between preoperative and 1** ^st^ ** year (P value)**	**0.001**	**0.001**	-
Number of medications	**Mean± SD**	**Mean± SD**	**P value**
** Preoperative **	3.1 ± 0.60	2.8 ± 0.81	0.196
** 1** ^st^ ** month **	0.47 ± 0.64	0.57 ± 0.85	0.544
** 3** ^rd^ ** month **	0.79 ± 1.0	0.79 ± 0.89	0.59
** 6** ^th^ ** month**	0.86 ± 1.1	1.2 ± 1.1	0.9
** 1** ^st^ ** year **	1.1 ± 1.1	1.1 ± 1.0	0.817
** Difference between preoperative and 1** ^st^ ** year (P value)**	**0.001**	**0.0017**	-
Complications and 2^ry^ Surgery	**Percentage (%)**	**Percentage (%)**	
** Bleb failure (n** ^br^ ** of eyes)**	47%	53%	**0.045**
** Needling, 5FU injection and 2** ^ry^ ** trabeculectomy (n** **of eyes)**	47%	57%	**0.04**

Data are presented as mean ± SD. Abbreviations: PXG: Pseudoexfoliative glaucoma; MMC: mitomycin C; IOP: intraocular pressure; 5FU: 5-fluorouracil; 2ry: secondary; n: number; SD: standard deviation; %: percentage; P values < 0.05 are in bold.

**Figure 4:**
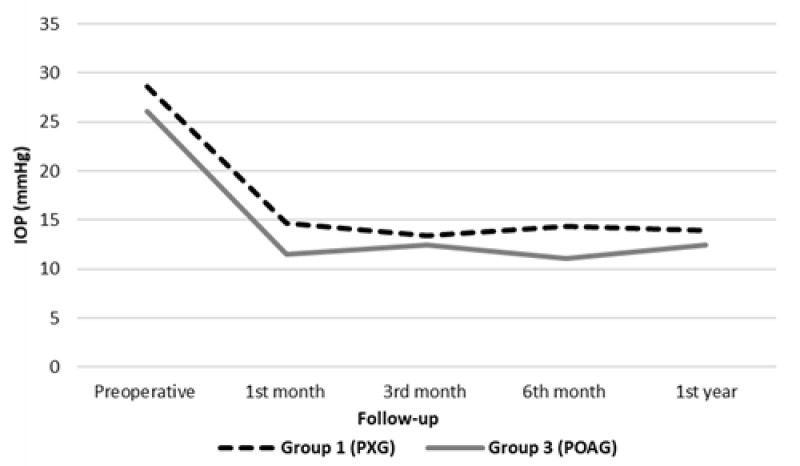
Evolution of Intraocular Pressure (IOP) in MMC Groups. Abbreviations: PXG: Pseudoexfoliative glaucoma; MMC: mitomycin C; POAG: Primary open angle glaucoma.

**Figure 5:**
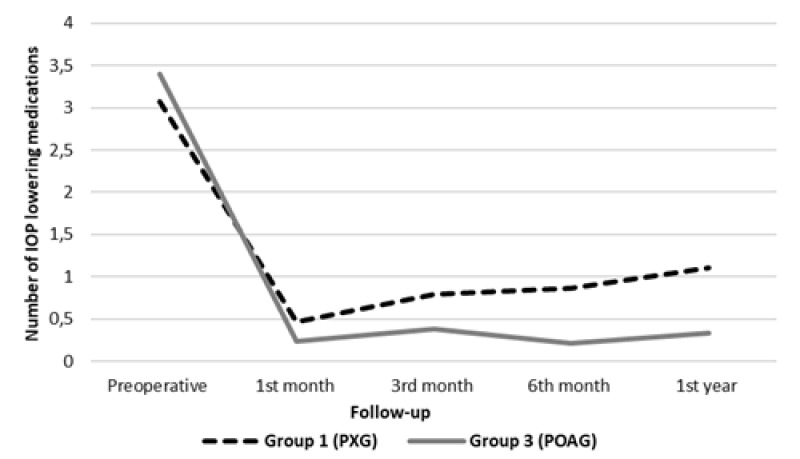
Evolution of Number of Medications in MMC Groups. Abbreviations: PXG: Pseudoexfoliative glaucoma; MMC: mitomycin C; POAG: Primary open angle glaucoma.

PHASE II – Comparison between MMC groups (group 1 – PXG, and group 3 – POAG group), Table 4: 

1. Evolution of IOP (Fig 4): in group 3, mean ± SD of IOP was 26.1 ± 6.5 mmHg before surgery, 11.5 ± 4.6 mmHg at the 1st month, 12.5 ± 5.1 mmHg at the 3rd month, 11.1 ± 3.9 mmHg at the 6th month and 12.5 ± 4.0 mmHg at the 1st year. Group 1 had higher IOP values at all stages of the follow-up, but a statistically significant difference in IOP reduction was found between the two groups only at the 6th month after surgery (P = 0.045). The difference between preoperative IOP and postoperative IOP at the 1st year was statistically significant in both groups (with equal significance, P = 0.001).

2. Evolution in the number of medications (Fig 5): in group 3 the mean ± SD of the number of medications was 3.4 ± 0.76 before surgery, 0.24 ± 0.56 at the 1st month, 0.38 ± 0.89 at the 3rd month, 0.21 ± 0.58 at the 6th month and 0.33 ± 0.89 at the 1^st^ year. Despite a slightly lower number of medications in the preoperative period (without a significant difference), group 1 had a higher number of medications at all stages in the postoperative period, with statistically significant differences at 6^th^ month and at the 1^st^ year (P = 0.048 and P = 0.036, respectively). The difference between preoperative IOP and postoperative IOP at the 1^st^ year was statistically significant in the both groups (P = 0.001 in group 1 and P = 0.006 in group 3).

3. Bleb failure: group 1 had a higher prevalence of bleb failure than group 3 (47% versus 18%, P = 0.02).

4. Additional surgical procedures performed after trabeculectomy: additional surgical procedures were performed more often in group 1 (47% versus 6%), with a statistically significant difference (P = 0.013).

**Table 4:** Comparison Between Groups 1 and 3 (group 1-PXG with MMC, and group 3-POAG with MMC)

	Group 1 (PXG)	Group 3 (POAG)	Significance of the difference
IOP	Mean ± SD	Mean ± SD	P value
** Preoperative **	28.6 ± 5.4	26.1 ± 6.5	0.175
** 1** ^st^ ** month **	14.7 ± 5.8	11.5 ± 4.6	0.151
** 3** ^rd^ ** month **	13.4 ± 4.0	12.5 ± 5.1	0.329
** 6** ^th^ ** month **	14.4 ± 5.3	11.1 ± 3.2	**0.045**
** 1** ^st^ ** year**	13.9 ± 3.9	12.5 ± 4.0	0.3
** Difference between preoperative and first year (P value)**	**0.001**	**0.001**	-
Number of medications	Mean ± SD	Mean ± SD	
** Preoperative**	3.07 ± 0.59	3.4 ± 0.76	0.114
** 1** ^st^ ** month**	0.47 ± 0.64	0.24 ± 0.56	0.195
** 3** ^rd^ ** month**	0.79 ± 0.97	0.38 ± 0.89	0.111
** 6** ^th^ ** month**	0.86 ± 1.1	0.21 ± 0.58	**0.048**
** 1** ^st^ ** year**	1.1 ± 1.1	0.33 ± 0.89	**0.036**
** Difference between preoperative and first year (P value)**	**0.001**	**0.006**	-
Complications and 2^ry^ Surgery	Percentage (%)	Percentage (%)	P value
** Bleb failure (n** ^br^ ** of eyes)**	47%	18%	**0.02**
** Needling, 5FU injection and 2** ^ry^ ** trabeculectomy (n** ^br^ ** of eyes)**	47%	6%	**0.013**

**Abbreviations: PXG: Pseudoexfoliative glaucoma; POAG: Primary open angle glaucoma; IOP: intraocular pressure; 5FU: 5-fluorouracil; 2ry: secondary; n** ^br^ **: number; SD: standard deviation; %: percentage; P values < 0.05 are in bold.**

## DISCUSSION

In this study, measured outcomes in the PXG group without MMC application were tendentially worse compared with the PXG group with MMC application and with the POAG group, supporting the hypothesis of PXG being a relevant risk factor for bleb failure. The differences between these groups are consistent with the fact that PXG is a more aggressive type of glaucoma, due to a high inflammatory profile. This profile is thought to contribute to a worse prognosis with higher IOP, and the necessity of more medication and secondary surgical procedures [1-6]. Therefore, surgical MMC application can probably improve the outcome of trabeculectomy in PXG patients, as it reduces episcleral–conjunctival fibrosis through potent antifibrotic action [10-14].

There are several studies about trabeculectomy outcomes and the potential risk factors for trabeculectomy failure in POAG, as well as different surgical protocols on MMC application, which vary in exposure time, MMC concentration, delivery vehicle and the size or shape of the sponge [1, 10, 15-17]. However, there is no standard protocol and there are few studies regarding MMC application in PXG patients [1, 10, 15-17]. Furthermore, not all studies comparing the outcomes of trabeculectomy between POAG and PXG include the application of MMC [1]. PXG which could be unilateral at presentation is not yet a standard risk factor for trabeculectomy failure, so that it alone justifies the application of MMC to reduce that risk. Most studies regarding the surgical trabeculectomy outcomes in PXG and POAG patients were not statistically different, reporting similar surgical outcomes in both types of glaucoma, contrary to theoretical expectations. The trabeculectomy outcomes also do not seem to differ between phakic or pseudophakic eyes, as well as do not seem to be affected by the antifibrotic applied. Additionally, no surgical complications seem to be exclusive to PXG patients. However, most of the authors treat trabeculectomy with steroids for a longer duration of time to reduce the risk of bleb failure, considering the greater inflammatory profile of the PXG eyes [6, 19-23].

In the comparison with the POAG surgery with MMC, the results of our study are similar to those of Lim et al. [1] as the authors did not find a statistically significant difference between PXG and POAG groups regarding preoperative IOP and number of medications and both found a statistically significant difference in the number of medications at the last moment of follow-up. They showed similar success rates for trabeculectomy with MMC in PXG and POAG patients at the 1st year, but poorer long-term IOP control from the second postoperative year. Ehrnrooth et al. [24] reported that complete success rates (without medication) were signiﬁcantly better in a POAG group than a PXG group, but this diﬀerence was not statistically signiﬁcant when qualiﬁed success (with a single topical medication) was included; furthermore, they did not use any adjunctive antiﬁbrotics. Also, Landers et al. [25] in a 20-year follow-up study of trabeculectomy reported that PXG is more likely than other types of glaucoma to progress to blindness, with a statistically significant difference [1]. On the other hand, Landa et al. [26] reported that the success rate was 83.6% in a PXG group and 83.7% in a non-PXG group at a mean of 12.6 months after phacotrabeculectomy with MMC. Studies such as those of Jerndal et al. [21] and Popovic et al. [27] also reported similar results between POAG and PXG white patients. 

Therefore, this issue remains controversial. Studies have applied different definitions of success rate and different MMC protocols, making comparisons between studies difficult. Other parameters as the follow-up period, ethnic diﬀerences between patients, the genetic intra and inter-ethnic variance and environmental influences may also justify the difference of results between studies [1-6]. The follow-up period is an essential factor to consider, as surgical success rate after trabeculectomy is known to decline over time, as shown in the study of Lim et al., but several studies have reported surgical success rates for PXG in a relatively short follow-up period [1]. With respect to other parameters, the prevalence of PXS varies among diﬀerent populations and patterns of PXS-associated LOXL1 gene variants in Asian diﬀer from whites [1, 4, 7], as shown, for example, by different results between the study of Lim et al., performed in Asian patients and the other studies mentioned, performed in whites. PXS is currently considered the single most important risk factor for the development of open-angle glaucoma [6, 28] and in some populations (as populations of the Baltic countries, the Mediterranean region and the Arab population), the frequency of PXS associated with secondary open-angle glaucoma may be even higher than the POAG [5, 6, 28-30]. In this context, it is worth noting that the current study was performed in a Portuguese population (belonging to the Mediterranean region). However, the pathogenesis of PXS and PXG is not completely understood, given that variables such as genetic risk variance and environmental influences remain poorly characterized [3-6]. Therefore, further research is essential. Some of the studies suggesting that the trabeculectomy outcomes in PXG are worse than POAG, as the current study, speculate that the primary cause may be the more severe destruction of the blood-aqueous barrier after trabeculectomy in PXG. This may lead to marked postoperative inﬂammation, fibrous reaction and subsequent ﬁltering bleb scarring and failure [1, 5, 6]. Konstas et al. [22] reported a more common ﬁbrous response after trabeculectomy in PXG eyes, and Nguyen et al. [31] reported more severe destruction of the blood-aqueous barrier in PXG eyes through quantification of aqueous ﬂare in the anterior chamber. Another mechanism that may play an important role is continuous deposition of extracellular materials in the anterior segment of PXG eyes after surgery, contributing to the aqueous outﬂow decrease. Thus, the inflammatory response associated with the mentioned mechanisms might lead to worse postoperative trabeculectomy outcomes in PXG [1, 5, 6].

Aspects that strengthen this study are the fact that surgeries were performed by the same experienced surgeon (thus reducing interindividual surgical and postoperative biases); comparison with the POAG group; and the goal of improving clinical practice through discussion of a controversial issue of great clinical importance. Additionally, this study raises the importance of performing a continuous postoperative follow-up. This study also had some limitations, such as the retrospective design, the relatively small sample and the one-year follow-up period. Thus, we suggest future investigations with longer follow-up period and prospective design to achieve more conclusive results. 

## CONCLUSIONS

PXG may be a risk factor for filtration bleb failure and should be considered for inclusion in surgical protocols of MMC application. As PXG is considered a more aggressive type of glaucoma, associated with a higher inflammatory profile, improvement of surgical protocols may contribute to improved clinical practice. More studies, featuring a larger sample and longer follow-up period, are necessary to validate this hypothesis and to support the eventual revision of surgical protocols. 

## References

[1] Lim SH, Cha SC (2017). Long-term Outcomes of Mitomycin-C Trabeculectomy in Exfoliative Glaucoma Versus Primary Open-Angle Glaucoma. J Glaucoma.

[2] Hollo G, Katsanos A, Konstas AG (2015). Management of exfoliative glaucoma: challenges and solutions. Clin Ophthalmol..

[3] Eyewiki - American Academy of Ophthalmology Pseudoexfoliative Glaucoma 2017. http://eyewiki.aao.org/Pseudoexfoliative_Glaucoma.

[4] Zenkel M, Schlotzer-Schrehardt U (2014). Expression and regulation of LOXL1 and elastin-related genes in eyes with exfoliation syndrome. J Glaucoma.

[5] Aboobakar IF, Johnson WM, Stamer WD, Hauser MA, Allingham RR (2017). Major review: Exfoliation syndrome; advances in disease genetics, molecular biology, and epidemiology. Exp Eye Res..

[6] Desai MA, Lee RK (2008). The medical and surgical management of pseudoexfoliation glaucoma. Int Ophthalmol Clin.

[7] Sagong M, Gu BY, Cha SC (2011). Association of lysyl oxidase-like 1 gene polymorphisms with exfoliation syndrome in Koreans. Mol Vis.

[9] Koliakos GG, Konstas AG, Schlotzer-Schrehardt U, Hollo G, Mitova D, Kovatchev D (2004). Endothelin-1 concentration is increased in the aqueous humour of patients with exfoliation syndrome. Br J Ophthalmol.

[10] Lu LJ, Hall L, Liu J (2018). Improving Glaucoma Surgical Outcomes with Adjunct Tools. J Curr Glaucoma Pract.

[11] Meyer LM, Graf NE, Philipp S, Fischer MT, Haller K, Distelmaier P (2015). Two-year outcome of repeat trabeculectomy with mitomycin C in primary open-angle and PEX glaucoma. Eur J Ophthalmol.

[12] Hirunpatravong P, Reza A, Romero P, Kim EA, Nouri-Mahdavi K, Law SK (2016). Same-site Trabeculectomy Revision for Failed Trabeculectomy: Outcomes and Risk Factors for Failure. Am J Ophthalmol..

[13] Fontana H, Nouri-Mahdavi K, Lumba J, Ralli M, Caprioli J (2006). Trabeculectomy with mitomycin C: outcomes and risk factors for failure in phakic open-angle glaucoma. Ophthalmology.

[14] Vaz FT, Henriques S, Lopes AS, Silva D, Mota M, Lisboa M (2017). Qual o papel dos anti-metabolitos como adjuvantes intra e pós-operatórios? Glaucoma: Perguntas Frequentes.

[15] Chen PP, Yamamoto T, Sawada A, Parrish RK, 2nd, Kitazawa Y (1997). Use of antifibrosis agents and glaucoma drainage devices in the American and Japanese Glaucoma Societies. J Glaucoma.

[16] Singh K (1997). Antimetabolite application: science or voodoo?. J Glaucoma.

[17] Maquet JA, Dios E, Aragon J, Bailez C, Ussa F, Laguna N (2005). Protocol for mitomycin C use in glaucoma surgery. Acta Ophthalmol Scand.

[18] European Glaucoma Society (2014). Secondary Glaucomas. Terminology and guidelines for glaucoma.

[19] Budenz DL, Pyfer M, Singh K, Gordon J, Piltz-Seymour J, Keates EU (1999). Comparison of phacotrabeculectomy with 5-fluorouracil, mitomycin-C, and without antifibrotic agents. Ophthalmic Surg Lasers.

[20] Fontana H, Nouri-Mahdavi K, Caprioli J (2006). Trabeculectomy with mitomycin C in pseudophakic patients with open-angle glaucoma: outcomes and risk factors for failure. Am J Ophthalmol.

[21] Jerndal T, Kriisa V (1974). Results of trabeculectomy for pseudo-exfoliative glaucoma A study of 52 cases. Br J Ophthalmol.

[22] Konstas AG, Jay JL, Marshall GE, Lee WR (1993). Prevalence, diagnostic features, and response to trabeculectomy in exfoliation glaucoma. Ophthalmology.

[23] Scott IU, Greenfield DS, Schiffman J, Nicolela MT, Rueda JC, Tsai JC (1998). Outcomes of primary trabeculectomy with the use of adjunctive mitomycin. Arch Ophthalmol.

[24] Ehrnrooth P, Lehto I, Puska P, Laatikainen L (2002). Long-term outcome of trabeculectomy in terms of intraocular pressure. Acta Ophthalmol Scand.

[25] Landers J, Martin K, Sarkies N, Bourne R, Watson P (2012). A twenty-year follow-up study of trabeculectomy: risk factors and outcomes. Ophthalmology.

[26] Landa G, Pollack A, Rachmiel R, Bukelman A, Marcovich A, Zalish M (2005). Results of combined phacoemulsification and trabeculectomy with mitomycin C in pseudoexfoliation versus non-pseudoexfoliation glaucoma. Graefes Arch Clin Exp Ophthalmol.

[27] Popovic V, Sjostrand J (1999). Course of exfoliation and simplex glaucoma after primary trabeculectomy. Br J Ophthalmol.

[28] Ritch R (1994). Exfoliation syndrome-the most common identifiable cause of open-angle glaucoma. J Glaucoma.

[29] Kozobolis VP, Papatzanaki M, Vlachonikolis IG, Pallikaris IG, Tsambarlakis IG (1997). Epidemiology of pseudoexfoliation in the island of Crete (Greece). Acta Ophthalmol Scand.

[30] Aasved H (1979). Prevalence of fibrillopathia epitheliocapsularis (pseudoexfoliation) and capsular glaucoma. Trans Ophthalmol Soc U K.

[31] Nguyen NX, Kuchle M, Martus P, Naumann GO (1999). Quantification of blood--aqueous barrier breakdown after trabeculectomy: pseudoexfoliation versus primary open-angle glaucoma. J Glaucoma.

